# Occupational Risks in Occupational Therapy Service Learning: A Single-Site “Fear Factor” Study in South Africa

**DOI:** 10.1155/2020/4746813

**Published:** 2020-04-28

**Authors:** Deshini Naidoo, Pragashnie Govender, Stephanie Nicole Naidoo, Naledi Ngubane, Zamankosi Nkosi, Aziza Mulla

**Affiliations:** Discipline of Occupational Therapy, School of Health Sciences, University of KwaZulu-Natal (Westville Campus), South Africa

## Abstract

**Background:**

Service-learning constitutes the main practical component of an undergraduate health profession training programme. However, limited exploration of the potential occupational risks that students face during their service-learning placement is noted in the literature.

**Aim:**

This study in South Africa explored occupational risk factors as reported by occupational therapy students whilst engaged in service-learning.

**Methods:**

In this explorative qualitative study, purposeful homogenous sampling was used to recruit third and fourth level occupational therapy students who completed a Bachelor of Occupational Therapy degree. Three focus groups were conducted with seventeen students who voluntarily participated. Audio-recorded data were transcribed and analysed thematically using deductive reasoning.

**Results:**

Several concerns over potential occupational risks were reported, namely, anxiety over contracting infectious diseases, concerns around musculoskeletal injuries due to manual handling requirements, and inappropriate behaviour of clients, which made the students feel unsafe. Concerns around the implementation of infection control measures, the lack of resources allocated to infection control at some service-learning sites, as well as the coping strategies used during service-learning were highlighted.

**Conclusion:**

Varied occupational risk factors during service-learning were reported by students. These insights into the perceived occupational risk factors can be translated into actionable strategies to improve the preparation of health science students for service-learning, including coping skills to deal with the demands of service-learning.

## 1. Introduction

A student's professional development is shaped by a complex academic and experiential journey that seeks to transition them from theory-driven students to skilled health professionals [1–6]. Students' exposure to suitably designed service-learning experiences is essential for the acquisition of key graduate attributes required for professional practice [3–6]. Service-learning thus constitutes the core practical component of an undergraduate health profession programme. In occupational therapy, students have to complete a minimum of 1000 hours in service-learning, often at public-sector hospitals, nongovernmental organisations (NGOs) or in the community, to meet the international [2] and local [7] regulatory body's minimum standards of training.

Service-learning is defined by the World Federation of Occupational Therapists as “the time students spend interpreting specific client-related factors and their relationship to health and well-being, establishing and evaluating therapeutic and professional relationships, implementing an occupational therapy process (or some aspect of it), demonstrating professional reasoning and behaviours, and generating or using knowledge of the contexts of professional practice with and for real live people” [2, 8].

There is consensus that service-learning orientates students to professional practice by facilitating learning of their profession, within actual practice settings, whilst concurrently serving as a health professional [3, 5, 6, 9]. During service-learning, students are provided with an opportunity to enact graduate attributes such as problem-solving skills, lateral thinking, and the ability to be reflexive, flexible, and capable of ethical behaviour, all of which are considered essential for professional practice [10, 11]. Service-learning occurs in variable sites and often occupational therapy students are likely to work in underresourced and remote areas during their placements. Moreover, due to extreme staff shortages within certain sites within the public sector, students are often required to work independently as a primary service provider, especially in their final year of study.

Whilst the role of service-learning is acknowledged, there is an ongoing debate as to the factors that influence the acquisition of professional knowledge and the manner in which students are acculturated to deliver an occupational therapy service [1–7, 9, 11]. There also remains a paucity of research on occupational therapy students' perspective of their experiences during service-learning and the consequent influence on professional development, especially within a South African context.

The need for this study arose from anecdotal reports of students who felt unprepared for the challenges they experienced during service-learning especially the reality of the world of work in constrained contexts such as that of South Africa. In one study in the country [12], the authors reported that occupational therapy students in South Africa face poverty, violence, crime, abuse, and uncertainty due to constantly changing institutional and societal structures during their service-learning placements. It was therefore essential to understand the concept of risk perception and occupational risk as related to students in this study.

Risk perception is the ability of a person to assess the likelihood of a specified type of accident or negative outcome occurring as well as evaluating the consequences of the negative outcome against possible gain [13–15]. This complex evaluation of potential negative outcomes is based on the individual's frame of reference, level of knowledge, and relevant social and cultural values and ideology [13–16]. There are several factors that influence risk perception [15]. At a macrolevel, the institution/leadership and/or supervisor's attitude and commitment toward safety influence health workers' risk perception [15–18]. Health workers are less likely to engage in high-risk behaviour if there was a high emphasis on safe work procedures [15, 17, 18]. At a mesolevel, peers and community pressure influence risk perception. Pressure from peers both in the workplace and outside can influence health workers to take risks. For example, a new health worker can choose to use shortcuts with job tasks after observing more seasoned workers, or refuse to use personal protective equipment to avoid being perceived as weak [19, 20]. At a microlevel, the health workers' level of knowledge regarding the situation, feeling of personal control, and level of optimism bias, that is, the belief that the negative event is less likely to happen to them, influences their risk perception. Cooper [19] and McCool and colleagues [21] found that individuals took more risks when they underestimated their susceptibility and the severity of an event, overestimated the level of protection they had in place, and their ability to cope with risk, especially if their knowledge regarding the situation is perception rather than fact.

Occupational risks, in the context of this study, refer to the potential barriers that hinder student's learning experiences and performance during their service-learning placement. Concha-Barrientos and colleagues [22] define occupational risk factors as a chemical, physical, environmental, biological, human, or other agent that could cause harm to a person in the workplace and is potentially modifiable. Due to the nature of health care service delivery, health care workers face the possibility of being exposed to workplace occupational risk factors, which include, amongst others, exposure to infectious materials, which includes body substances, contaminated medical supplies and equipment, contaminated environmental surfaces, or contaminated air and harmful/aggressive behaviour from patients [23]. Needle prick injuries, musculoskeletal injuries, allergens, viruses, bacteria, and parasites are further occupational risks that could pose a threat to health care workers' health and well-being [24]. Health care workers and students are exposed to physical risk factors due to physically demanding tasks, e.g., manual patient handling that requires lifting and transfer of heavy patients, bending, and twisting [25, 26]. Additionally, long working hours, incorrect working postures, and poorly applied ergonomics have been acknowledged as factors that contribute to the development of musculoskeletal injuries [25–27]. Musculoskeletal injuries in health care workers are seen seven times more than that of any other industry due to the need for clients to be positioned, lifted, and transferred to the bed, chair, and toilet [25].

McCarthy et al. [28] reported a high tuberculosis (TB) transmission rate between health care workers and patients in the South African context. The high human immune-deficiency virus (HIV) and the TB epidemic affecting the majority of the South African population could be a contributing factor to the TB transmission rate. Health care workers have two to three times the TB infection rate of nonmedical workers [29, 30]. Students in the health care industry and related occupations face risks of occupational exposure to bloodborne pathogens, including HIV, hepatitis B virus, hepatitis C virus, and other potentially infectious materials [31, 32]. Irrespective of whether the student is infected or not, there is a period of anxiety, guilt, worry, or fear [24]. These emotions limit a student's ability to holistically assess and implement intervention with patients who have contagious infections or illnesses. Therefore, students become less hands-on with their patients and skills are not fully developed, which negatively impact their service-learning and future service delivery [24].

Violence against health care workers in a Palestine study shows that more than 50% of the health care workers are victims of violence such as verbal abuse, threatening behaviours, and physical assault [33]. During service-learning, students in the South African context may experience verbal abuse or threatening behaviours when they are placed in the primary health care facilities or when treating psychiatric patients [8]. Preconceived ideas and fears are established due to the negative stigma attached to persons suffering from psychiatric or physical illnesses in clinical placements. These impact on students' willingness to actively engage in therapy sessions with their clients. WHO reports that workplace violence is a phenomenon which affects every country, every workplace, and every health care professional group [30]. Workplace violence is complex and one of the most dangerous hazards faced by health care workers. There is an increased risk for exposure to violence, by patients who are under the influence of substances, such as drugs and alcohol, metabolic disorders, trauma, psychosis, personality disorders, or by the stress of patients and relatives who face long waiting hours and overcrowding [23]. Moreover, workplace factors that may contribute to student stress include dealing with life-threatening illnesses, injuries such as blunt force trauma or knife wounds, demanding patients, being overworked, a lack of human resources, the lack of specialized equipment, the hierarchy of authority, lack of control, lack of participation in planning and decision making, and patient deaths [25].

### 1.1. Aim of the Study

This study sought to explore occupational therapy students' perception of occupational risk and their experience of occupational risks in service-learning sites. By gaining greater understanding into student's risk perception, health professional educators may be more suitably positioned to raise awareness and ensure safe and conducive learning environments for students under training.

## 2. Methodology

### 2.1. Research Team and Reflexivity

Four authors were responsible for conducting the interviews in this study (SNN, NN, ZN, and AM). At the time of the study, these four final year female students were part of the student cohort that was invited to participate in this study. This team, hence, enjoyed having established rapport with participants as well as having occupied a hybrid stance as a student-researcher. Interest in the research was, therefore, shared collectively and the team members were able to suspend their judgements as student-researchers by an audit trail, peer debriefing with DN and PG, as well as by investigator triangulation (all authors).

### 2.2. Study Design

This study followed an exploratory qualitative design, which was used to explore and understand the meaning that students ascribed to the issue of occupational risk factors in their service-learning placements [34, 35].

### 2.3. Participant Selection

All senior-level occupational therapy students (*n* = 30 third year and *n* = 39 fourth-year students) were eligible to participate in the study. Purposeful homogenous sampling was adopted to select the final sample [35]. The study sought to pool students of comparable service-learning backgrounds and experiences to contribute in focus groups on their perceptions and experiences of occupational risk factors during service-learning [34, 35]. Participants were recruited via email request. The final sample comprised 17 students who volunteered to participate.

All of the student participants in this study were female (100%; *n* = 17). The mean age of the students was 21.5 years. The majority of the students were in the third year of study (64.7%; *n* = 11) with 35% (*n* = 6) in the fourth year of study. The majority of the students (64.7%; *n* = 11) had completed three service-learning blocks with 29.4% (*n* = 5) having completed six service-learning blocks and one student 5.9% (*n* = 1) having completed eight service-learning blocks (Table 1).

### 2.4. Study Location

The study was located at a higher institute of learning in one province of South Africa. Within this province, there is only one training institute that offers training for health professionals, and was hence suitable for the recruitment of occupational therapy students who would have potentially experienced occupational risk factors while engaging in service-learning at hospitals and clinics.

### 2.5. Data Collection

Data were collected via three focus group discussions that spanned between 45 to 60 minutes each with a maximum of six participants. In each of the focus groups, two of the four authors were present (SNN, NN, ZN, and AM). This team underwent training in focus group etiquette and piloted the focus group schedule on another group of students (not part of this study). Following informed consent, discussions were audio-recorded.

### 2.6. Data Analysis

The audio-recorded focus groups were transcribed verbatim. Content analysis using deductive reasoning ensured. Data were manually coded and organized. Initial coding assisted in the organization of the data into segments prior to themes [36]. Four of the authors initially coded the data (SNN, NN, ZN, and AM). This was then categorised via peer debriefing with DN and PG. All authors were responsible for the reduction of the data into themes and verification of themes against the original spoken words to ensure the credibility of the findings. The themes were represented using a narrative passage and interspersed by verbatim quotes. Within each of the focus groups, the two facilitators paraphrased and summarised responses to ensure that this was captured authentically. This together with feedback following the data analysis assisted in ensuring respondent validation and member checking in the study.

### 2.7. Ethical Considerations

The authors were guided by the Declaration of Helsinki [37] in ensuring that study participants were informed volunteers in the study, and that their integrity was respected by ensuring confidentiality.

## 3. Findings

Three themes emerged from the analysis of the data and are presented with associated subthemes and supported by verbatim quotes.

### 3.1. Theme 1: Anxiety (“It's like you're thrown into the deep end without being given a chance to swim”)

Within this theme, students expressed concerns over the risk of infection, risk of musculoskeletal injury, as well as concerns over inappropriate behaviours of clients.

### 3.2. The Risk of Infection: “I don't want to touch her ever again”

Students reported having a fear of contracting infection and diseases during their service-learning blocks due to the hands-on nature of work that occupational therapy entails when working with clients.

“A kid came and he held my hand and he had full on scabies. Now I'm paranoid now that I'm going to get scabies.” – Participant 1, 22 years, Level 4 student

### 3.3. Musculoskeletal Injury Risks: “For a week we could not use our arms”

Students expressed concern regarding patient transfers and the negative effects that it had on them. Negative effects included back pain, inability to complete their service-learning blocks, and fear of dropping a client.

“Moving and mobilising patients during transfers and using my physical strength because I'm not a fit person and I'm not strong enough and then I also got a chronic illness which exacerbates every time I do physical activity.” – Participant 2, 21 years, Level 4 student

### 3.4. Inappropriate Behaviour of Clients: “How do you handle someone who is doing that to you”

Students expressed their concern for inappropriate behaviour of clients during service-learning which made them feel uncomfortable and sometimes unsafe.

“This guy was making very inappropriate moves and you can't do anything about it. You have to act like professional, so it puts your personal space and attitude towards the patient at risk.” – Participant 3, 20 years, Level 3 student

## 4. Theme 2: Infection CONTROL (“I just wanted to protect myself from all the germs”)

### 4.1. Implementation of Infection Control Measures

Students expressed a lack of enforcement of infection control protocol during service-learning which created negative emotions and fear.

“I've been to wards where they say you don't have to wear a TB mask but then I see doctors wearing TB masks and then I'm not wearing one and I start getting all like itchy and weird.” – Participant 4, 20 years, Level 3 student.

Students communicated that the sound implementation of infection control protocols in certain service-learning venues assisted them to be at ease and not constantly have to worry about their health.

“At my venue, they emphasise safety protocol quite a lot. Like masks and the gloves and then make sure you are washing your hands all the time. So for me, it made it a lot easier the fact that I was wearing proper masks, gloves and there were sanitizers available.” – Participant 5, 20 years, Level 3 student

### 4.2. Limitations of Infection Control

It appeared that whilst infection control was largely beneficial during service-learning, it came with its limitations, particularly in establishing rapport with clients and in an undermining of students' capabilities.

“Even though we should be wearing masks and aprons and things like that, it gets a bit difficult to communicate with your patients and then you lack with, you start to lack with that rapport building.” – Participant 2, 21 years, Level 4 student

## 5. Theme 3: Coping through Service-Learning

### 5.1. Internal Spiritual Conflicts: “I feel like that's hypocrisy”

Students reported feelings of discontentment due to the nature of work that service-learning required. They felt that their values were not accommodated during service-learning which created a sense of uneasiness.

“I feel like that's hypocrisy because I don't believe in it but I'm supposed to educate you on it.” – Participant 3, 20 years, Level 3 student

### 5.2. “At the end of the day, you have to study”

Students verbalised that because service-learning is an integral component of the undergraduate degree, they were willing to face any occupational risks in order to complete their degree.

“But at the end of the day, it's … you have to study, you have to finish it. So, no matter even if you are scared, you going to have to go in there and do what you have to do.” – Participant 2, 21 years, Level 4 student

Students verbalised that to successfully complete service-learning blocks, they utilised various forms of support which assisted them to remain resilient despite the occupational risks that they experienced.

“For me, my coping strategy is definitely talking to my colleagues when I'm having a bad day. And they would actually motivate me to carry on, actually get through the day. Literally moral support.” – Participant 6, 23 years, Level 4 student

### 5.3. Motivators for Participation “I got this genuine love for people, I love doing OT”

Despite the occupational risks that students faced during service-learning, they mentioned their appreciation for the degree and concern for clients, which motivated them to participate.

“I've got this genuine love for people so regardless of the fact that they are HIV positive or negative, you've got TB, all that. So I was like it's fine, she's got it but then I still have to show affection to her.” – Participant 7, 20 years, Level 3 student

## 6. Discussion

A number of occupational risks encountered by occupational therapy students during service-learning within the public health care sector were noted in this study. The study comprised only female volunteers. The sense of anxiety emanating from the tension between the students' inherent desire to protect themselves from perceived risks versus their need to develop their professional practice abilities were clearly observed. These are outlined in the discussion that follows.

On a macro- and mesolevel, adherence and commitment to infection control protocols or lack thereof in service-learning sites, especially those that were under-resourced, elicited anxiety in students. This served as a barrier to the students' maximal participation in service-learning, due to feelings of being unprotected and uncomfortable whilst working with patients with infectious illnesses. Infection control protocols are part of the espoused health and safety policies in the public health sector however there are varied levels of implementation. Keorekile [26] suggests that once a student is exposed to an infectious agent, irrespective of whether the student is infected or not, there is a period of anxiety, guilt, worry, or fear. These findings are corroborated by international literature on the negative impacts of a lack of infection control [24, 29, 31]. In this study, students highlighted that a negative experience caused by exposure to bodily fluids resulted in one student contracting gastroenteritis. This experience negatively influenced the students' consequent behaviour (volition towards participating in service-learning) as a result of the perceived threat of being exposed to biological factors. Their hands-on approach towards clients diagnosed with infectious illnesses was altered and replaced with new boundaries that defined their engagement with patients. Authors within the available literature acknowledge students who become less hands-on with their patients that result in their skills not being fully developed, which inevitably negatively influences the outcomes expected of the service-learning placement [15, 24]. The students highlighted that they experienced more positive experiences in service-learning sites where more effective infection control measures were implemented. These measures resulted in reduced risk perception as experienced by students. Students felt that they were able to have more direct contact with patients as they were not afraid of contracting illnesses because they were adequately protected, thus, impacting on their overall hands-on intervention with clients.

On a microlevel, anxiety, tensions between one's belief system and focus on the outcome of grades and not risks, as well as musculoskeletal risks were noted. Students drew attention to the increased anxiety and stress due to the demands of the academic programme and hospital service delivery, which prevented students from performing efficiently during service-learning. The increased anxiety and stress levels were said to also impact their quality of life and balance. Both Chhabra [23] and Gorman and colleagues [38] studies support these findings, as the impact of workplace and personal stress on health care workers has been noted to lead to lifestyle imbalance. More positively, the findings indicated that students found various mechanisms to cope with stress and anxiety that were elicited by their participation in service-learning. Students stated that their peers, family, and supervisor formed part of their support system during service-learning which created a counter-balance for the anxiety and stress and a source of comfort. The presence of a support system was noted to positively influence the students' overall performance during service-learning. These findings are supported by authors within the available literature who acknowledge the role of coping mechanisms as a means to reduce stress by problem-solving and developing abilities to manage stress, which will benefit students on a personal level and with the delivery of patient intervention [39–42].

Spiritual beliefs posed as both a barrier and a facilitator to student-learning. Students face the dilemma of completing the requirements of service-learning despite the tension it poses with respect to their own religious and/or spiritual beliefs. Students face multiple internal conflicts to ensure that there is responsible service delivery. The reward of achieving the desired outcomes of service-learning and their desire to gain success in the degree outweighs the perceived risk posed by their spiritual conflict.

Students reported exposure to physical risk factors such as musculoskeletal pain. Alnaser [25] and Ogunlana and colleagues [27] studies support the findings in that health care workers develop musculoskeletal injuries as they are required to lift and carry patients. Students reported that they experienced musculoskeletal strain when they are required to transfer patients from various platforms such as from the bed to the wheelchair. Certain factors such as the increased body weight of the clients and inappropriate environmental equipment such as nonadjustable beds place strain on students, resulting in back pain and musculoskeletal strains. Kitaneh and Hamdan [33] indicated that health care workers are exposed to verbal or physical abuse and threatening behaviours. Students elaborated on the inappropriate behaviour displayed by clients such as articulation of inappropriate comments, which contribute towards students feeling uncomfortable and unsafe. This perceived lack of safety negatively affected the students' ability to build rapport with their clients and deliver the quality of therapy required.

It was evident from the study that the completion of the degree influenced the student's motivation to participate during service-learning. The students drive to fulfill all requirements of service-learning was fuelled by the need to complete their degree despite the tension created by the perceived occupational risk. This may not always create a conducive environment for learning or developing professional reasoning skills, as students may avoid situations where they perceive high occupational risk factors, or only perform when their academic or clinical supervisors are present which may lead to ethical breaches.

### 6.1. Limitation of the Study

The findings are only applicable to the occupational therapy students and cannot be generalized to all health science students, as this was an explorative study. Additionally, the sample was exclusively female, which is representative of the demographics of the student cohort; however, this may have influenced the findings.

## 7. Conclusions

Students are faced with multiple occupational risk factors during the course of their service-learning including physical, biological, and environmental risks. In this study, these occupational risks appeared to influence students' ability to complete their roles as a student therapist thereby affecting their performance capacity. The implications for educators and programmes during service-learning include, firstly, the need to raise awareness and provide more effective orientation programmes for infection control procedures, manual handling, and dealing with difficult patients prior to students commencement of their service-learning, and secondly, to embed more formalised peer support systems and processes to reflect on critical incidents as a part of service-learning to improve the students' ability to cope with the concomitant demands and improve their ability in gaining professional knowledge. This would potentially mitigate the negative effects of occupational risk factors as identified in this particular study.

## Figures and Tables

**Table 1:** Demographics of students (*n* = 17).

		% (*n*)
Gender	Female	100 (17)

Age	20 years	47 (8)
	21 years	24 (4)
	22 years	12 (2)
	23 years	18 (3)

^1^Race	African	24 (4)
	White	12 (4)
	Indian	53 (9)

Year of study	3^rd^ year	65 (11)
	4^th^ year	35 (6)

Completed service learning blocks	3 blocks	65 (11)
	6 blocks	29 (5)
	8 blocks	6 (1)

Most challenging block	Acute setting	94 (16)
	Chronic setting	6 (1)

Classification according to the Population Registration Act No 30 of 1950, South Africa.

## Data Availability

The qualitative data used in this study is available of request.

## Abbreviations

HIV: Human immune-deficiency virus.
TB: Tuberculosis.
NGO: Nongovernmental organisation.
HCP: Health care practitioner.
WHO: World Health Organisation.
